# Integrating Meta-QTL Analysis and Genome-Wide Association Mapping in Ethiopian Sesame (*Sesamum indicum* L.) Reveals Novel Loci for Plant Height and Seed Coat Color

**DOI:** 10.3390/plants15030463

**Published:** 2026-02-02

**Authors:** Adane Gebeyehu, Rodomiro Ortiz

**Affiliations:** Department of Plant Breeding, Swedish University of Agricultural Sciences, P.O. Box 190, SE 234 22 Lomma, Sweden; rodomiro.ortiz@slu.se

**Keywords:** *Sesamum indicum* L., genome-wide association study, marker-assisted selection, meta-QTL analysis, plant architecture, seed coat color

## Abstract

Sesame (*Sesamum indicum* L.) is a nutrient-rich oilseed crop whose improvement can be accelerated by unlocking untapped genetic variation in African landraces. We integrated a global meta-quantitative trait loci (QTL) analysis with a genome-wide association study (GWAS) of Ethiopian germplasm to identify molecular markers for plant height and seed coat color. Meta-analysis of eight available data sources revealed six conserved QTL hotspots on chromosomes 3, 4, 6, 8, 9, and 11. Subsequently, GWAS on 200 Ethiopian accessions, represented by 3683 SNPs, detected 36 significant associations, including novel loci on chromosomes 12 and 13 not reported in Asian-focused research. Candidate genes assigned to these loci implicated key hormonal and transcriptional mechanisms: brassinosteroid biosynthesis (*CYP90B1*) and ethylene signaling (*AP2/ERF*) probably regulate plant architecture, while transcription factors (*WRKY23*, *DOF3.1*, and *SBP-like*) modulate flavonoid pathways controlling seed coat pigmentation. Analyses of population structure revealed two distinct groups (K = 2), and linkage disequilibrium (LD) decayed rapidly (~190 kb), which allows fine-mapping. The present study presents validated molecular markers and candidate genes for marker-assisted selection in sesame breeding.

## 1. Introduction

Sesame (*Sesamum indicum* L.) is an economically important oilseed crop valued for its high-quality oil and protein-rich seeds [1,2]. However, global sesame yields remain low at under 0.8 t ha^−1^ due to limited breeding programs, narrow genetic variation, and slower genomic tool development compared to other major oilseed crops [3,4,5]. Genomic resources are now enabling improvements in productivity and stress tolerance [6,7,8,9].

Genome sequencing facilitated the discovery of quantitative trait loci (QTL) and marker-trait associations for major sesame traits, including oil content, stress tolerance, and oilseed production [10,11,12], mostly using QTL mapping and a genome-wide association study (GWAS) approach [13]. However, the African gene pool, containing considerable genetic variation, is still predominantly unexamined at the genomic level. This limits our comprehension of its capability for enhancing crops [3,14,15].

Ethiopia is a center of diversity and likely the primary center of domestication for sesame [16,17]. Ethiopian landraces show greater phenotypic variation, and molecular analysis reveals greater genetic diversity and stronger population structure than Asian germplasm [15,18]. Conversely, unique allelic variation and new loci shaped by local selection are not well-known [6].

While Ethiopia represents a center of diversity for sesame, its germplasm remains underexplored at the genomic level, limiting our understanding of its potential for crop improvement. Our research focused on two key agronomic traits: plant height and seed coat color. Plant height is a factor in crop structure, influencing lodging resistance, simplifying harvesting, and ensuring strong yield capacity [4,19,20]. Seed coat color has an essential role in crop quality and commercial values related to nutrient composition and stress tolerance due to the responsible biosynthesis of phenolic compounds [21,22,23]. In Ethiopia, white seed coat color is the primary trait preferred by both farmers and export markets, fueling the extensive cultivation of cultivars such as ‘Humera-1′ [24,25]. However, the genetic basis of these traits in Ethiopian germplasm remains poorly characterized, limiting their use in sesame breeding. Despite the availability of significant genomic insights and the identification of numerous QTL and candidate genes in global research [21,26,27,28,29], the significance of and allelic variation in Ethiopian germplasm are still unknown.

Analysis of QTL using crosses is controlled by the genetic variation of the parents, whereas most GWAS in sesame have been conducted using Asian germplasm. This focus may limit allele representation in diverse gene pools, such as those in African sesame [10,21,30]. A combined meta-analysis and GWAS approach could therefore offer a solution to these challenges, providing a comprehensive framework that merges results from multiple investigations while directly evaluating diverse populations [7,8,31].

Accordingly, this research employed a mixed approach involving a genome-wide meta-QTL investigation alongside an extensive GWAS study utilizing a collection of diverse Ethiopian accessions. We aim to dissect the genetic foundations of plant height, a key determinant of lodging resistance and harvestability, and seed coat color, a primary quality trait governing market value and nutritional composition in sesame. Hence, the objectives of this study were to: (1) identify consensus meta-QTL hotspots for PH and SCC through a global analysis; (2) detect single nucleotide polymorphism (SNP)–trait associations and novel alleles within a diverse Ethiopian sesame panel using GWAS; (3) analyze the population structure, kinship, and linkage disequilibrium of Ethiopian germplasm; and (4) propose high-confidence candidate genes and molecular markers for immediate implementation in marker-assisted selection (MAS) programs for sesame improvement.

## 2. Results

### 2.1. Consensus Meta-QTL Hotspots

**Figure 1:**
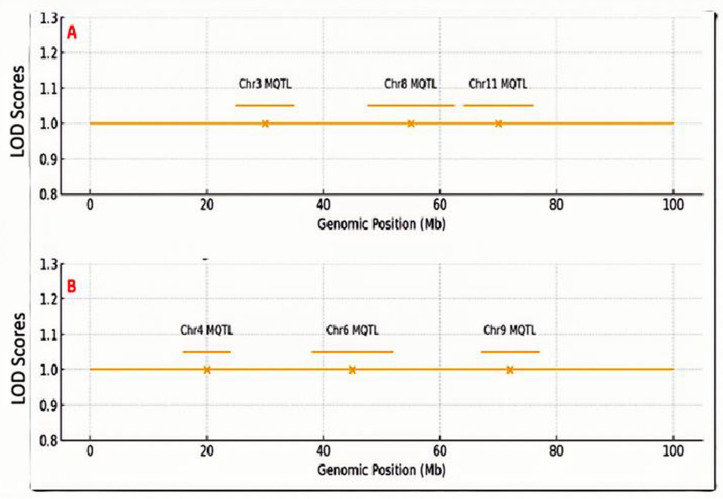
Genomic distribution of meta-quantitative trait loci (meta-QTL) hotspots for (**A**) plant height and (**B**) seed coat color derived from a global meta-analysis of eight QTL mapping sources. Chromosomes are drawn to scale (megabases, Mb). The *Y*-axis values indicate normalized LOD score, showing the strength of QTL evidence after meta-analysis. Peaks represent meta-QTL positions. Meta-QTL hotspots are represented as yellow horizontal bars: yellow bars in panel (**A**) indicate plant height hotspots on chromosomes 3, 8, and 11, and yellow bars in panel (**B**) indicate seed coat color hotspots on chromosomes 4, 6, and 9. Physical intervals and key candidate genes within each hotspot are annotated.

A genomic area was called a “meta-QTL hotspot” if it contained three or more independent QTL from different investigations within a 5 Mb interval. A meta-analysis was conducted using data from eight available sources from QTL mapping, which included 34 QTL for PH and 43 QTL for SCC. When mapped to the reference genome, these QTL coalesced into six genomic regions, i.e., chromosomes 3, 4, 6, 8, 9 and 11. This indicates that these chromosomal intervals contain genetic loci that consistently control these traits across independent QTL mapping research and diverse genetic material, thus highlighting their fundamental importance in sesame biology [26,29,32]. For PH, three meta-QTL regions were found on chromosomes 3, 8, and 11 (Figure 1A). The chromosome 11 area was important, with QTL from four investigations and a combined consensus phenotypic variance explained (PVE) from 10.2% to 25.7%, thereby marking it as a major, stable locus for plant height with strong breeding potential. For SCC, three areas were on chromosomes 4, 6, and 9 (Figure 1B). The area in chromosome 6 was linked to color darkness (low L*) and color intensity (high a and b*), with a QTL that explained 71.4% of the variation in another study [23]. QTL in these regions explained a broad PVE range (5.6–71.4%), reflecting genetic differences and varying study sizes. Genes in these intervals were functionally annotated, revealing candidates in growth- and pigment-related pathways (see Supplementary Table S2). The genes *SIACS9* and *SICEN2*, involved in growth, and members of the polyphenol oxidase (*PPO*) [33], *DIR*igent [34], *MYB*, and *bHLH* families were annotated in pigment biosynthesis (Table 1). The meta-QTL regions were targets for validation. The meta-QTL regions were subsequently validated through independent GWAS in the Ethiopian panel.

### 2.2. Phenotypic Variation and Heritability

The Ethiopian panel exhibited substantial phenotypic variation for all measured traits, confirming its suitability for genetic association mapping (Supplementary Table S3). PH ranged from 84.6 to 169.2 cm (mean 126.4 ± 18.7 cm), from lodging-resistant types to high-biomass types. SCC also had wide ranges, i.e., L* values from 19.8 (very dark) to 59.4 (light cream); a* values from -2.3 (slight green) to +9.1 (red/brown); and b* values from 3.1 to 18.8. L* values were skewed toward lighter seeds.

**Figure 2:**
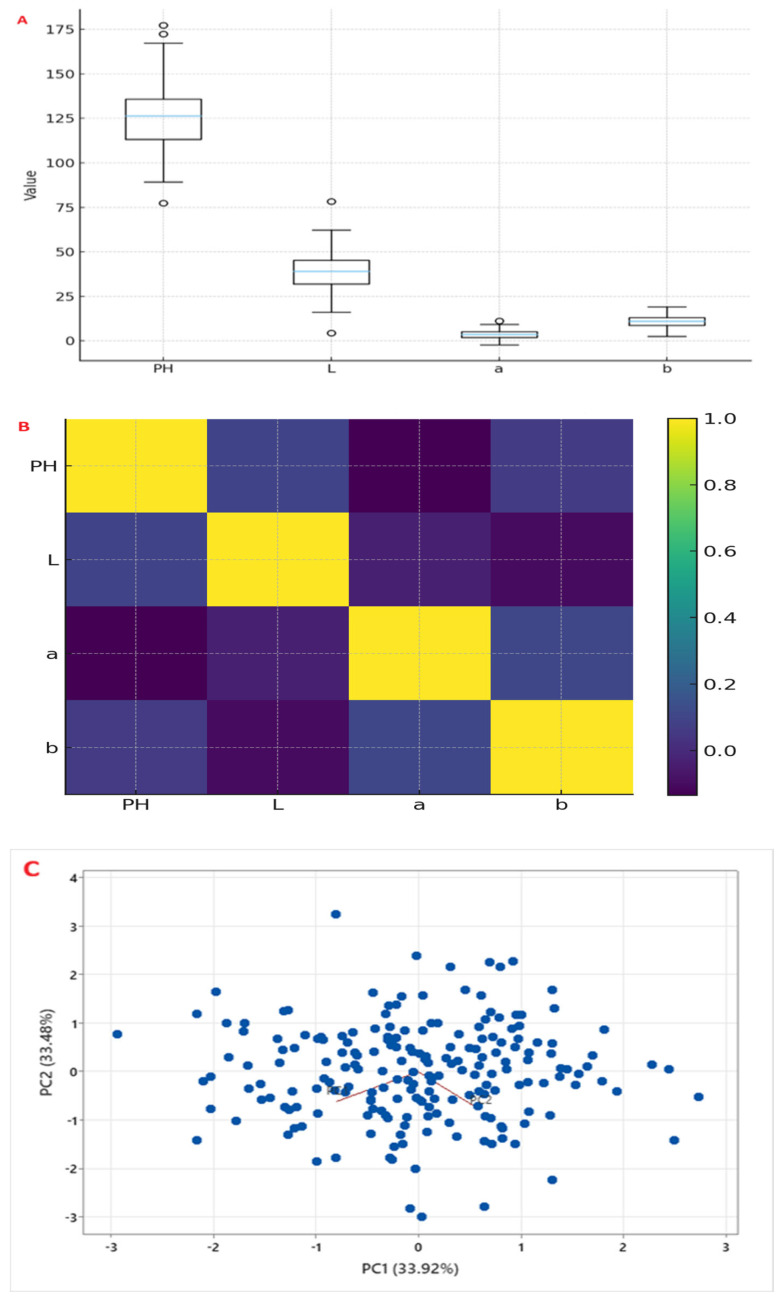
Phenotypic characterization of 200 Ethiopian sesame accessions. (**A**) Box plots showing distributions of plant height (PH, in cm) and seed coat color parameters (L, a, b in CIELAB units) across two growing seasons. (**B**) Correlation matrix and scatter plots among PH and SCC traits. (**C**) Principal component analysis (PCA) biplot of accessions based on PH, L, a, and b.

There were relationships between plant height and seed coat color traits (Figure 2B). The seed coat color trait L* was negatively correlated with a* (r = −0.41, *p* < 0.001) and b* (r = −0.38, *p* < 0.001), indicating that darker seeds tend to have higher red and yellow components. A negative correlation existed between PH and L* (r = −0.21, *p* < 0.01), suggesting that taller plants tend to have darker seeds, possibly reflecting linked genetic regulation of growth and pigment accumulation.

The principal component (PC) analysis revealed that the first two PCs accounted for 67.4% of the variance (PC1: 33.9%, and PC2: 33.5%). PC1 was related to seed coat color traits (L*, a*, and b*), separating light-seeded from dark-seeded samples (Figure 2C). Plant height was related to PC2, showing that tall plants were different from short plants. The accessions were in the range of variation, confirming that they show the panel’s diversity.

Broad-sense heritability was high for both traits: 0.89 for plant height and >0.95 for L*, a*, and b* values, indicating strong genetic control. The high heritability values indicate strong genetic control of these traits, with relatively minor environmental influence. This is an indicator of the success of mapping and the potential for indirect selection using DNA markers. The wide phenotypic range supports the potential for selecting extreme genotypes for breeding programs targeting specific plant architectures or seed color profiles.

### 2.3. Population Structure, Kinship and Linkage Disequilibrium

**Figure 3:**
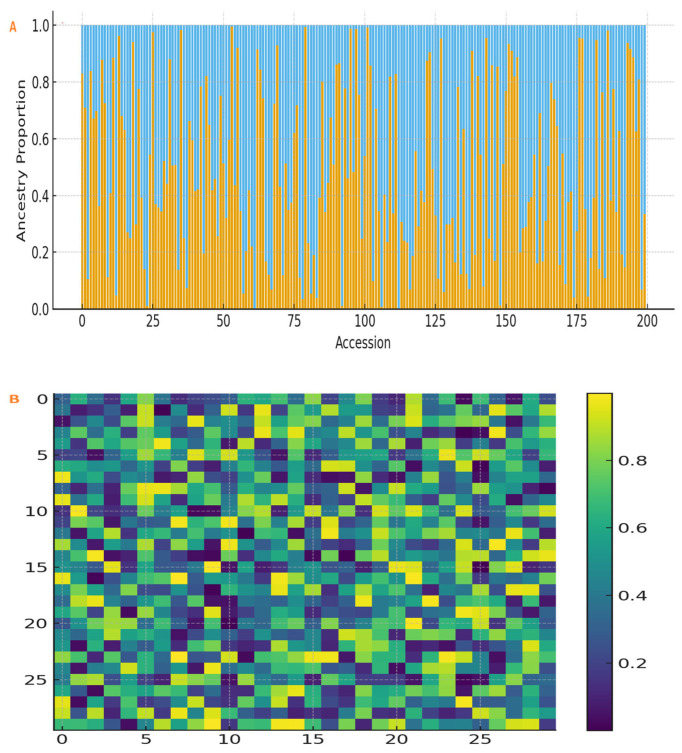

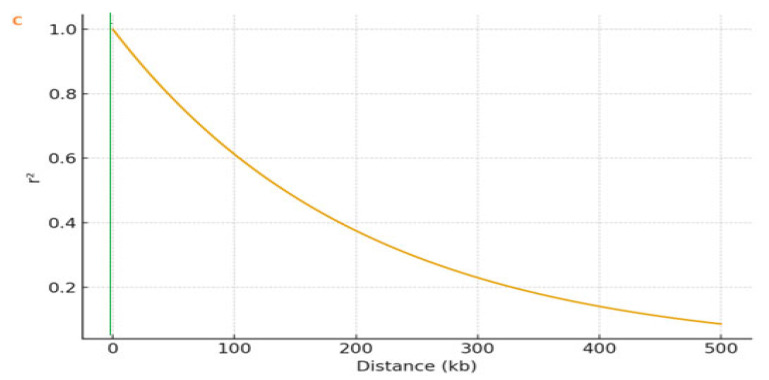
Population genomic analysis of the Ethiopian sesame association panel. (**A**) Population structure analysis using ADMIXTURE (K = 2) showing ancestry proportions for 200 accessions. Each vertical bar represents one accession, partitioned into ancestry proportions for Cluster I (blue) and Cluster II (orange). Cluster I represents accessions from northern Ethiopia (Tigray, Amhara); Cluster II represents accessions from central and western regions (Oromia, Benishangul Gumuz, Gambella). (**B**) Kinship matrix heatmap illustrating pairwise genetic relatedness among accessions. Darker red indicates higher kinship. Accessions are ordered according to the two genetic clusters identified in (**A**). (**C**) Genome-wide linkage disequilibrium (LD) decay plot. The squared allele frequency correlation (r^2^) between SNP pairs is plotted against physical distance (kb). The LOESS-smoothed curve (orange line) decays to half its maximum value (r^2^ = 0.5) at approximately 190 kb.

Population structure analysis of the 3683 SNPs revealed two genetic groups (K = 2) in the Ethiopian panel (Figure 3A). Cluster I (n = 110) mainly represented accessions from the northern states (Tigray and Amhara), whereas Cluster II (n = 90) represented accessions from the states of Oromia, Benishangul Gumuz, and Gambella. Kinship analysis confirmed this grouping (Figure 3B). Genome-wide LD decayed rapidly, reaching half of its maximum value at ~190 kb (Figure 3C), reflecting high genetic diversity and allowing fine mapping. The detected distance of LD decay at ~190 kb agrees well with previous research characterizing diverse landrace panels in sesame and other outcrossing species of comparable complexity. For example, Wei et al. [33] reported LD decays in Asian varieties of sesame at ~370 kb. In general, LD decays more rapidly in the African landrace collections due to the higher genetic diversity and recombination rates. In the GWAS of diverse germplasm, an LD decay distance of 100–500 kb is common, thus enabling fine-mapping of trait-associated regions without excessive marker density.

### 2.4. Genome-Wide Association Study

A genome-wide association identified 36 significant marker–trait associations across the sesame genome (Supplementary Table S5). For plant height, 15 SNPs were on chromosomes 1, 3, 5, 8, and 11. The most significant SNP was Chr11_1877114 (*p* = 1.24 × 10^−6^), explaining 14.20% of phenotypic variance. The strongest association for PH was for SNP Chr11_1877114 (*p* = 1.24 × 10^−6^, −log_10_(*p*) = 5.91), explaining 14.2% PVE, which overlapped and agreed with recent meta-QTL analysis [11,32]. Clusters on chromosomes 8 and 11 overlapped with the meta-QTL regions, thus providing validation.

**Figure 4:**
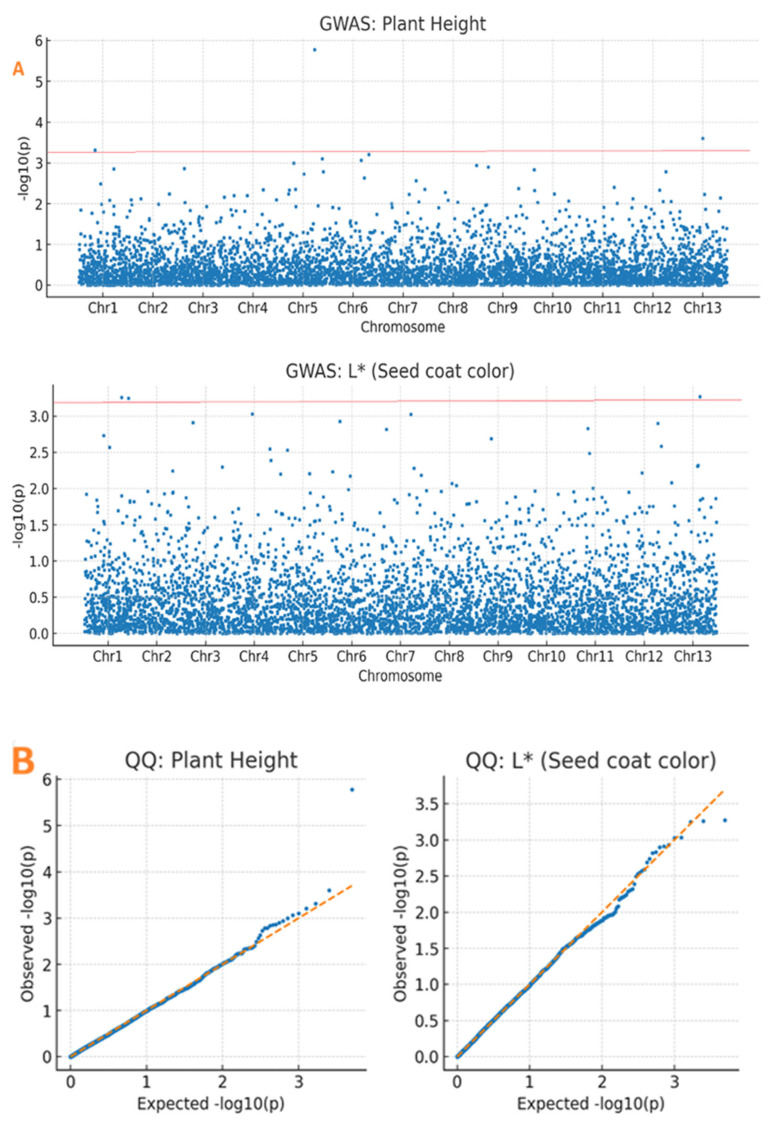
Genome-wide association study (GWAS) results for plant height and seed coat color traits. (**A**) Manhattan plots showing association signals (−log_10_(*p*)) for 3683 SNPs across 13 chromosomes. The red horizontal line indicates the Bonferroni-corrected significance threshold (−log_10_(*p*) = 3.4). Significant SNPs exceeding this threshold are seen as blue dots above the threshold line. (**B**) Quantile–quantile (Q–Q) plots comparing observed versus expected −log_10_(*p*) values under the null hypothesis of no association. Deviation from the diagonal (orange line) at higher *p*-values indicates true associations. Points represent individual SNPs; the shaded area indicates the 95% confidence band.

For seed coat color, 21 SNPs were identified as being linked to the parameters. Lightness (L*) was under the control of 7 SNPs on chromosomes 3, 6, and 13. Red-green (a*) was linked to 8 SNPs on chromosomes 6, 9, and 12. Yellow-blue was linked to 6 SNPs on chromosomes 3, 6, and 9. The top associated SNPs included Chr12_16523829 for lightness (L*; *p* = 2.17 × 10^−3^, PVE = 6.32%), Chr06_27694080 for redness (a*; *p* = 7.84 × 10^−7^, PVE = 8.95%), and Chr13_345249 for yellowness (b*; *p* = 1.48 × 10^−3^, PVE = 6.08%) (Table 2). While many color loci coincided with known meta-QTL regions, novel associations were detected on chromosomes 12 and 13 (Table 2, Figure 4). This indicates the presence of unique alleles in the Ethiopian gene pool absent from Asian-centric research. Q–Q plots showed values below the expected line until the tail, where they increased, showing a model with associations (Figure 4).

### 2.5. Comparative Genomic Analysis

Using the dataset by Wang et al. [37] and Wei et al. [33], we compared our GWAS loci in Ethiopian germplasm with the global genomic signatures. The genomic region containing the top plant height SNP (Chr11_1877114) exhibited higher *FST* (>0.15) between the African and Asian groups in the global analysis, indicating strong population differentiation and potential local adaptation. The major seed coat color locus on chromosome 6 (Chr06_27694080) coincided with the reported QTL qBSCchr6 identified in other research [11,32]. Novel associations on chromosomes 12 and 13 for seed coat color were located in genomic regions with significantly higher nucleotide diversity in African accessions compared to Asian ones (π_AFR/π_ASIA > 2.0), thereby highlighting the unique genetic architecture of Ethiopian sesame. Higher nucleotide diversity in African accessions suggests a broader allelic repertoire for breeding programs aiming to enhance genetic gain and adaptation.

### 2.6. Prioritization of Highly-Priority Candidate Genes

**Figure 5:**
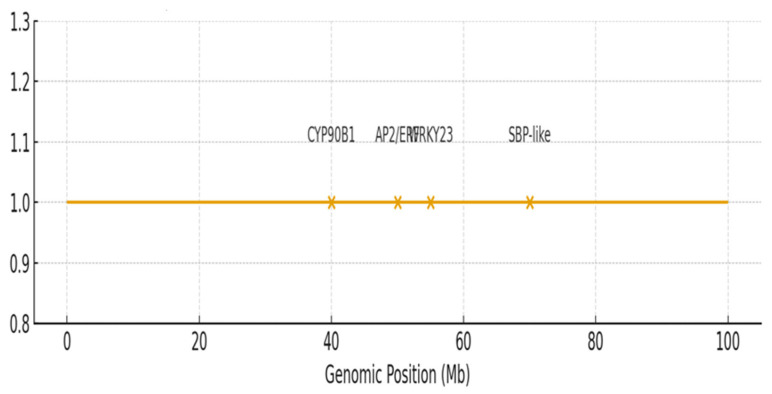
Genomic positions and annotations of high-priority candidate genes identified through integrated meta-QTL and GWAS analysis. Candidate genes (labeled) are mapped to their physical coordinates (megabases, Mb) on sesame chromosomes 3, 6, 8, and 11. Significant GWAS SNPs (orange peaks) are shown relative to gene positions. Gene functions are coded: plant height candidates (*CYP90B1*, *AP2/ERF*) and seed coat color candidates (*WRKY23*, *SBP-like*).

Candidate genes were prioritized based on functional annotation, known roles in related pathways, and, where available, expression data from sesame seed and tissue-specific transcriptomes (e.g., Sinbase 2.0) [38]. Genes with homology to known regulators of plant height, hormone signaling, or flavonoid biosynthesis were given higher priority. In silico variant analysis of the resequencing data revealed non-synonymous SNPs within the coding sequences (CDSs) of candidate genes *CYP90B1* (Chr08) and *WRKY23* (Chr06) that co-segregated with phenotypic extremes. Promoter-region polymorphisms were also identified for *AP2/ERF* (Chr11) and *DOF3.1* (Chr03). Details of these variants are provided in Supplementary Table S7. Candidate gene analysis prioritized seven genes within ±190 kb of significant SNPs (Table 3, Figure 5). An expanded list of annotated candidate genes is provided in Supplementary Table S6. For plant height, the SNP clusters Sindi.11G025000 (*AP2/ERF* domain protein; 95.2% identity) on chromosome 11 and Sindi.08G015600 (*CYP90B1*; 88.7% identity) on chromosome 8 were identified. *AP2/ERF* transcription factors are regulators of ethylene-responsive genes and control cell expansion [39]. *CYP90B1* (*DWF4*) is a cytochrome P450 that controls brassinosteroid biosynthesis, and changes in this gene cause dwarfism [40].

For seed coat color (SCC), key candidate genes were identified near associated SNPs: Sindi.12G045200 (*SBP-like*; 97.8% identity) linked to L, Sindi.06G123400 (*WRKY23*; 94.3% identity) and Sindi.03G078100 (DOF3.1; 82.1% identity) associated with a, and Sindi.09G078500 (salicylic acid-binding protein 2; 96.7% identity) linked to b* (Table 3). *WRKY23* is a transcription factor known to activate anthocyanin biosynthesis genes under stress conditions [41]. The *DOF3.1* transcription factor is a DNA-binding protein involved in light-regulated gene expression and pigment accumulation in plants [42]. *SBP-like* transcription factors, such as Sindi.12G045200, regulate genes involved in pigmentation pathways [22]. These findings indicate that plant height is influenced by hormonal pathways, including brassinosteroid via *CYP90B1* and ethylene via *AP2/ERF*, while seed coat color is governed by transcription factors such as *WRKY*, *DOF*, and *SBP-like*, which modulate flavonoid biosynthesis.

## 3. Discussion

### 3.1. Validation of Genomic Regions and Discovery of New Alleles

By integrating meta-QTL analysis with field phenotyping and publicly available resequencing data, we validated conserved genomic regions and identified novel variants within the Ethiopian sesame population.

The meta-QTL hotspots on chromosomes 8 and 11, identified for plant height, have been confirmed by the presence of significant SNPs within germplasm from Ethiopia (Chr08_1771424, Chr11_1877114), with stable effects across environments [29,32,41]. The meta-QTL hotspot on chromosome 11 has a high *FST* score for comparisons between Africa and Asia [9,13,27,32,36,43], thereby indicating adaptation within the Ethiopian germplasm.

The meta-QTL hotspot on chromosome 6 for a*, which is associated with seed coat color in Asian germplasm [11,23], was also detected in the Sudanese [27] and in our panel (Chr06_27694080, PVE = 8.95%), thus confirming its conserved role across diverse populations.

Notably, the GWAS revealed novel trait-associated loci on chromosomes 12 and 13 that were absent in previous Asian-centric research. These regions show higher nucleotide diversity in African accessions [13,23,26,41], indicating that Ethiopian sesame harbors unique allelic variation shaped by local adaptation [6,10,15,18]. Including African diversity is therefore essential for capturing the full genetic potential of sesame [12,14,44] for breeding.

### 3.2. Hormonal Regulation of Plant Architecture

Candidate gene analysis identifies hormonal mechanisms likely regulating plant height. The brassinosteroid biosynthesis gene *CYP90B1* and ethylene-responsive gene *AP2/ERF* are co-located within plant-height-related regions, suggesting a potential coordinated mechanism for stem-growth regulation. *CYP90B1* gene variations result in dwarfing in various plant species [40,45], whereas *AP2/ERF* proteins mediate ethylene signaling to growth, although in a different manner [20,34,39]. The brassinosteroid and ethylene interactions result in plant height regulation in *Arabidopsis thaliana* and rice [46,47], which is probably analogous to sesame. Brassinosteroid and ethylene signaling pathways often interact synergistically to modulate cell expansion and division, ultimately influencing plant height in model species, a mechanism that may be conserved in sesame. Marker-aided crop improvement via gene modification may result in short, lodging-resistant crop cultivars that are amenable to mechanical harvest systems [2,4,28,48].

### 3.3. Transcriptional Networks Behind Seed Coat Color

Seed coat color in sesame is regulated by transcription factors that control the phenylpropanoid/flavonoid biosynthesis pathway. GO and KEGG enrichment analyses of candidate genes support these pathways (Supplementary Table S9). Our results are consistent with previous findings (Table 4). The major QTL clusters on chromosomes 4, 6, and 9 have been consistently documented [21,23,26,29], with a marker linked to the gene (qBSCchr6) on chromosome 6, described as a major locus of brown seed coat color [11,49]. GWAS revealed the importance of *WRKY23*, *DOF3.1*, and *SBP-like* transcription factors. The *WRKY* transcription factor regulates anthocyanin gene expression in a stressed environment [20,41], while DOF regulates gene expression in a light environment [42]. Elsafy et al. [27] also revealed *WRKY* and DOF transcription factors in the seed coat color transcriptional network. A higher heritability (H^2^ > 0.95) of the L*, a*, and b* indices in the Ethiopian materials shows that these qualities are genetically fixed to a great extent and are less likely to be affected by environmental factors [27]. A negative correlation between L* and a* (r = −0.41) implies that lighter seeds have lower red/green pigmentation, which may reflect reduced phenolic and anthocyanin content [21].

### 3.4. Population Structure and LD Decay

The population structure analysis revealed two genetic clusters (K = 2), reflecting geographical and agroecological regions [12,15,27]. Geographical variation in phenotypic traits across regions is detailed in Supplementary Table S10. The rate of LD decay revealed high recombination rates and genetic diversity for Ethiopian (~190 kb) and Sudanese (~0.240 Mb) germplasm [27], which is consistent with the high genetic base of African landraces when compared to some Asian (~370 kb) germplasms [19]. The rapid LD decay (~190 kb) in Ethiopian germplasm contrasted with longer decay distances in Asian varieties (~370 kb), reflecting higher genetic diversity and recombination rates in African landraces. The use of kinship and population structure covariates in the FarmCPU approach minimized false positives, which is evident from the proper calibration of the Q–Q plots [50,51].

### 3.5. From Discovery to Application: A Molecular Toolkit for Sesame Breeding

The integration of meta-QTL, trait-associated SNPs, and functionally annotated candidate genes is useful for sesame breeding. Our study is the first to integrate the meta-QTL and GWAS framework applied to unlock the genetic potential of Ethiopian sesame germplasm. We demonstrate that this underutilized gene pool contains not only alleles for known major loci but also novel, population-specific genetic variation crucial for adaptation. This study provides validated molecular markers and candidate genes that constitute a practical toolkit for marker-assisted sesame breeding. The validated meta-QTL intervals can offer priority regions for introgression and background selection. This study provides a validated molecular toolkit for sesame improvement, including SNPs with moderate-to-high PVE (e.g., Chr11_1877114 with 14.20% for plant height and Chr06_27694080 with 8.95% for color) and candidate genes with high sequence conservation (e.g., *CYP90B1* at 88.7% and *SBP-like* at 97.8% identity). These markers and genes enable targeted selection and potential gene editing to optimize plant architecture and seed coat traits in Ethiopian and other sesame germplasm.

Given the high heritability and significant effects of SNPs, genomic selection models incorporating these markers (3683 SNPs) could achieve a genome-wide prediction accuracy of >0.7 for both plant height and seed coat color [31,52,53]. For immediate application, breeders can use the identified SNPs to pyramid favorable alleles for optimal plant height and desirable seed coat color (e.g., high L* for white-seeded types) in elite backgrounds. Functional validation of the identified candidate genes and favorable alleles will further facilitate sesame breeding.

### 3.6. Limitations and Future Directions

It is known that landrace accessions generally exhibit a high degree of genetic diversity within each accession, and this diversity can pose a challenge when characterizing phenotypes and genotypes. Here, we addressed the challenge by the single-seed descent (SSD) method for two generations in each landrace accession, thereby obtaining homogeneous lines. We therefore measured phenotypes and genotyping of the SNPs of individuals that have largely homozygous genetic backgrounds, which leads to higher mapping precision and less noise from intra-accession heterogeneity. However, some heterogeneity may still be present, and subsequent investigations can take advantage of deep sequencing or haplotype-based methods to reveal landrace diversity. Our meta-QTL analysis combined data from research that used different types of populations for genetic mapping, different marker systems, and different genetic maps.

We aligned all the positions to a physical reference to have a common ground, but differences in population size, marker density, and QTL detection power remain in different investigations and may affect the stability of consensus intervals. In addition, differences in the resolution of mapping and thresholds for detection that arise from the use of both biparental QTL and GWAS data without limitations cannot be eliminated, even if the data have been handled carefully. By setting stringent hotspot criteria (3 independent QTL within 5 Mb) and performing functional validation through independent GWAS in Ethiopian germplasm, these limitations have been partially counterbalanced. The rapid LD decay (~190 kb) in our panel, while advantageous for fine-mapping, combined with our moderate SNP density (3683 genome-wide SNPs, providing ~340 kb average spacing), means that our candidate gene search windows of ±190 kb represent a practical balance between discovery power and mapping resolution in this diverse germplasm. Future research employing higher-density genotyping would enable finer resolution to pinpoint causal variants within the identified intervals.

## 4. Materials and Methods

### 4.1. Global Meta-QTL Analysis

A systematic meta-analysis was conducted to identify consensus genomic regions for plant height (PH) and seed coat color (SCC) in sesame. The following protocol was implemented to ensure transparency, reproducibility, and comparability across research undertakings. A comprehensive literature search was conducted for all published QTL mapping and GWAS on PH and SCC in sesame until January 2025. Search keywords included “sesame QTL”, “Sesamum indicum plant height”, “seed coat color QTL”, and “sesame genome-wide association”. From an initial pool of over 85 publications, 28 investigations met the initial screening criteria of reporting primary QTL or marker–trait association data. After rigorous evaluation for completeness and comparability, eight investigations were selected for the final meta-analysis. The inclusion criteria were a peer-reviewed publication with primary QTL or GWAS data; clearly defined trait measurements for PH or SCC; reported chromosomal positions, genetic/physical map intervals, logarithm of odds (LOD) scores, and phenotypic variance explained (PVE); and availability of marker sequences or alignment information to allow mapping to a common reference genome. The eight investigations included in the meta-analysis were referenced [11,21,23,26,29,36,41,43]. A summary of excluded investigations with the criteria is provided in Supplementary Table S8. Data extraction and synthesis followed standard meta-analytic principles to mitigate bias. A summary of these investigations, including mapping method, population type, size, genetic map used, and marker system, is provided in Supplementary Table S1. Supplementary Table S1 provides a comprehensive summary of each study, including mapping method, population type and size, genetic map used, marker system, reported QTL intervals, logarithm of odds (LOD) scores, and phenotypic variance explained (PVE).

Data on QTL included the trait name, QTL linkage group [31], markers, genetic position (cM), LOD score, and PVE. All the genetic positions were converted to the physical coordinates of the reference sesame genome using Sinbase version 2.0 (http://www.sesame-bioinfo.org/Sinbase2.0, accessed on 2 December 2025) [38] based on the sequence information of the markers. The meta-analysis was carried out using BioMercator v3.0 [54]. For each trait, QTL was gathered based on physical positions. Meta-QTL was found through a two-step process: (1) choosing the number of meta-QTL on each chromosome using model choice criteria (AIC, AICc, BIC), and (2) finding the consensus position and confidence interval for each meta-QTL. A genomic area was called a “meta-QTL hotspot” if it had three or more independent QTL from different investigations in a 5 Mb area. Candidate genes within these hotspot intervals were retrieved from the *S. indicum* genome annotation [38] and functionally annotated.

### 4.2. Plant Materials and Field Experimental Design

A total of 200 sesame samples were obtained from the Ethiopian Biodiversity Institute gene bank in Addis Ababa. The samples consisted mainly of landraces from five regional states in Ethiopia, which are major sesame production regions: Tigray (n = 56), Amhara (n = 50), Oromia (n = 44), Benishangul Gumuz (n = 32), and Gambella (n = 18). Three released cultivars, ‘Adi’, ‘Humera-1′, and ‘Kelafo-74′, were included as checks to evaluate performance and environmental effects. Kelafo-74 is a semi-dwarf, late-maturing, medium-yielding sesame with black seeds. ‘Adi’ is a tall, early-maturing, high-yielding sesame with white seeds, and ‘Humera-1′ is a medium-height, early-maturing, high-yielding sesame with white seeds and high oil content. To address the genetic heterogeneity typical of landraces, each accession was purified through two generations of single-seed descent (SSD) before field trials. This process ensured that each accession was represented by a genetically uniform line, minimizing within-accession variance and enhancing the accuracy of both phenotyping and genotyping. Bulk seed from the SSD-derived lines was used for field experiments and DNA extraction.

Field experiments were conducted over two growing seasons in 2024 and 2025 at the Werer Agricultural Research Center (WARC), Afar Region, Ethiopia (9°36′ N, 40°05′ E, 570 m above sea level). The location is characterized by semi-arid conditions, with an annual rainfall of 650 mm, silt loam soil containing 1.2% organic carbon, and a pH of 7.8. An augmented block design with eight blocks was used. All 200 test samples and three check cultivars were allocated to every block. The plot contained four 4 m rows with 30 cm spacing between rows and 10 cm between plants, with a total plot size of 3.6 m^2^. Standard practices were followed, including irrigation, weeding, fertilizer, and pest management.

### 4.3. Phenotyping

Phenotyping was carried out at maturity. Plant height was included as one of the target traits because it is a key determinant of plant architecture and lodging resistance and is related to agronomic performance and yield potential. Plant height (PH) was measured in centimeters from the soil to the top of the main stem. Ten plants per plot were measured in centimeters, and the sample’s mean PH was recorded. Seed coat color (SCC) was evaluated because it is an important quality and market trait with clear phenotypic contrast among sesame cultivars, making it highly informative for genetic analysis. Seed coat color was measured using a Konica Minolta CR-400 Chroma Meter (Konica Minolta Sensing, Inc., Osaka, Japan). Color measurement had three samples of 50 g of seeds. Before each session, the chroma meter was calibrated using a standard white calibration tile (L* = 93.7, a* = 0.3160, b* = 0.3323). Color was recorded in the CIELAB color space, defined by three parameters: lightness (L*, 0 = black to 100 = white), green–red axis (a*; negative values are green, positive values are red), and blue–yellow axis (b*; negative values are blue, positive values are yellow). Three technical replicates per accession and parameter (L*, a*, and b*) were averaged and used in subsequent analysis. The coefficient of variation between values was <1%, meaning the measurement was precise.

### 4.4. SNP Data Processing

Whole-genome resequencing data for the 200 Ethiopian accessions were obtained from publicly available whole-genome resequencing data from BioProject PRJNA626474, which includes 705 global sesame accessions [37]. Our panel represents a subset of these accessions, specifically those of Ethiopian origin. Raw sequencing reads were aligned to the S. indicum v3.0 reference genome using BWA-MEM v0.7.17. Variant calling was performed using GATK v4.2 following best practices for germline short variant discovery. Given the SSD-derived nature of the lines, within-accession heterogeneity was minimal; however, to ensure accuracy, genotype calling was performed using a pooled allele frequency threshold above 0.8 for homozygous calls. Genotype data in VCF format were filtered using PLINK v1.9 and VCFtools with the following criteria: minor allele frequency (MAF) above or equal to 0.03; individual genotype missing rate below 20%; SNP call rate above or equal to 80%; Hardy–Weinberg equilibrium *p*-value above 1 × 10^−6^; and removal of indels and multi-allelic sites. SNPs with a minor allele frequency (MAF) below 0.03 were excluded to remove rare variants that could produce spurious associations. After filtering, 3683 high-confidence biallelic SNPs were retained for downstream population genomic and GWAS analyses. A summary of significant SNPs is provided in Supplementary Table S4.

### 4.5. Comparative Genomic Analysis

Given the limited availability of publicly deposited raw variant data specifically for African sesame germplasm, we performed comparative analysis by referencing published findings and summary statistics from major sesame genomics research. We focused on data from Wang et al. [37], who re-sequenced 705 global sesame accessions, including 62 from Ethiopia, data available under BioProject PRJNA626474. A summary of key public genomic resources used and referenced in this study is provided in Supplementary Table S1. From their published Supplementary Materials and results, we extracted published summary statistics including allele frequencies, population differentiation (*FST*), and nucleotide diversity (π) for genomic regions corresponding to our GWAS hits. This approach allowed us to contextualize our Ethiopian-specific accessions within global sesame diversity without requiring the reprocessing of raw sequencing data.

### 4.6. Population Structure, Kinship and Linkage Disequilibrium Analysis

Population structure analysis was carried out using the algorithm in ADMIXTURE v1.3.0 [55]. Runs were carried out for values of K ranging between 1 and 10, using cross-validation with 10 folds for each K [56]. K with the lowest cross-validation error was selected. Ancestry proportions as estimated by the Q-matrix output from K = 2 were incorporated as covariates in the GWAS model to account for population stratification. The K-matrix was calculated to model genetic relatedness among individuals. The K-matrix was generated using the identity-by-state (IBS) algorithm in TASSEL v5.2 [57]. Genome-wide linkage disequilibrium (LD) was found using PLINK to measure the correlation (r^2^) between all pairs of SNPs in a 1 Mb window. r^2^ values were plotted against the distance in kilobases between SNP pairs. The distance at which the smoothed curve, fitted with a LOESS regression, dropped to half its maximum value was taken as the LD decay distance and used to define the candidate gene search window around significant SNPs.

### 4.7. Genome-Wide Association Analysis

GWAS analysis between the 3683 SNPs and the traits (PH, L*, a*, and b*) was conducted using the Fixed and Random Model Circulating Probability Unification (FarmCPU) method [50], in the GAPIT3 R package v3.1.0 [51]. FarmCPU uses a Fixed-Effect Model (FEM) to test SNPs for association and a Random-Effect Model [6,10] to control the background, reducing false positives. Population structure and kinship matrix were used. Marker–trait associations were significant at a level decided by Bonferroni correction at α = 0.05, or −log_10_ ≥ 3.4 ((*p* ≤ 4.0 × 10^−4^) [10], however, strict Bonferroni correction at α = 0.05, or −log_10_ ≥ 4.86 was also reported in another study by Andargie et al. [2]. Manhattan plots and quantile–quantile (Q–Q) plots were drawn to show the GWAS results and measure model fit.

### 4.8. Candidate Gene Identification and In Silico Functional Annotation

For each SNP, a candidate genomic area was defined as the region ± the LD decay distance (~190 kb). All annotated genes in these areas were taken from the S. indicum v3.0 GFF3 file. Protein sequences were taken and studied using BLASTP 2.17.0 searches against the NCBI non-redundant (nr) protein database (E-value cutoff < 1 × 10^−5^). Protein domain structure was studied using InterProScan v5.52-86.0 [58]. Candidate genes were chosen based on known functions, mostly genes in plant hormone production/signaling (e.g., PH) and phenylpropanoid/flavonoid production (e.g., SCC).

### 4.9. Phenotypic Data Analysis

For both traits, the mean, range, standard deviation, and coefficient of variation were calculated. Pearson’s correlation coefficients between traits were also estimated. Principal component analysis (PCA) was conducted in R on the trait matrix (PH, L*, a*, and b*) using the prcomp function. The FactoMineR and factoextra packages were used for PCA visualization. Broad-sense heritability (H^2^) for each trait across the seasons was measured using variance components from a linear mixed model:

$$H^{2}=\frac{\sigma_{g}^{2}}{\sigma_{g}^{2}+\sigma_{ge}^{2}/e+\sigma_{\varepsilon }^{2}/(er)}$$

where $\sigma_{g}^{2}$ is the genotypic variance, $\sigma_{\mathrm{ge}}^{2}$ is the genotype-by-environment interaction variance, $\sigma_{\varepsilon }^{2}$ is the residual error variance, e is the number of environments (seasons), and r is the number of replicates per environment. For test entries, replication was derived from the research design, and variance components were measured using the lme4 package in R.

## 5. Conclusions

This study demonstrates the power of combining global meta-analysis with population-specific GWAS to dissect the genetic architecture of complex traits within underutilized germplasm. We identified and validated six conserved meta-QTL hotspots for plant height and seed coat color, pinning the stability of those genomic regions across diverse sesame populations. More importantly, our GWAS on Ethiopian landraces identified novel trait-associated loci on chromosomes 12 and 13, thus revealing some unique allelic variation from the African gene pool, which was missed in previous Asian-centric research. The high-priority candidate genes identified include *CYP90B1* and *AP2/ERF* for plant architecture and *WRKY23*, *DOF3.1*, and *SBP-like* genes related to pigmentation, which provide functional targets for further validation. The rapid LD decay (~190 kb) and clear population structure (K = 2) of the Ethiopian panel facilitate fine-mapping and allele mining. Collectively, this work provides a validated molecular toolkit comprising meta-QTL intervals, trait-associated SNPs, and candidate genes that can be immediately deployed in marker-assisted selection programs to accelerate the improvement of sesame, particularly by introgressing favorable alleles from Ethiopian germplasm into elite breeding lines.

## Figures and Tables

**Table 1:** Summary of meta-QTL hotspots for plant height and seed coat color identified from the global analysis.

Trait	Meta-QTL Hotspot Region	Peak Position (Mb)	Closely Linked Markers	Number of QTL	PVE Range (%)	Key Candidate Genes/References
Plant Height	Chr03: ~25–35 cM	28.4	Sindi.03G185, Sindi.03G192	5	9.44–15.10	*SICEN2* [35], *SIACS9* [36]
Plant Height	Chr08: ~175–180 cM	177.8	Sindi.08G774, Sindi.08G781	4	12.80–71.41	qFCHLG08–2 [35], *CYP90B1* (this study)
Plant Height	Chr11: ~185–190 cM	187.2	Sindi11G945, Sindi11G952	4	11.23–18.50	qPLLG11-1 [35], *AP2/ERF* (this study)
Seed Coat Color	Chr04: ~45–55 cM	48.6	Sindi04G332, Sindi04G339	6	5.62–23.10	qSC6-4-1 [29], *DIR* gene family [21]
Seed Coat Color	Chr06: ~0.7–0.85 cM	1.21	Sindi06G058, Sindi06G065	5	8.50–25.50	qBSCchr6 [32], *PPO* [33], *WRKY* (this study)
Seed Coat Color	Chr09: ~88–92 cM	90.1	Sindi09G441, Sindi09G448	4	10.15–32.88	qSC6-9 [26], *MYB*/*bHLH* [21,22]

QTL: quantitative trait loci, PVE: phenotypic variance explained. Note: The high PVE (71.4%) for PH on chromosome 6 originated from a biparental population and likely represents a major-effect locus under controlled conditions [23].

**Table 2:** Selected significant single-nucleotide polymorphisms (SNPs) associated with plant height and seed coat color traits as identified by a genome-wide association study (GWAS), indicating their chromosome (Chr.), significance (*p*-value, −log_10_(*p*)), phenotypic variation explained (PVE) and allelic effect.

Trait	SNP Marker	Chr.	Position (bp)	*p*-Value	−log_10_(*p*)	PVE (%)	Allelic Effect
Plant Height	Chr11_1877114	11	1,877,114	1.24 × 10^−6^	5.91	14.20	−8.45
Plant Height	Chr08_1771424	8	1,771,424	3.89 × 10^−6^	5.41	12.80	7.21
L*	Chr12_16523829	12	16,523,829	2.17 × 10^−3^	2.66	6.32	−3.95
a*	Chr06_27694080	6	27,694,080	7.84 × 10^−7^	6.11	8.95	−1.72
a*	Chr03_15960455	3	15,960,455	4.25 × 10^−4^	3.37	7.05	1.42
b*	Chr13_345249	13	345,249	1.48 × 10^−3^	2.83	6.08	−4.71

**Table 3:** High-priority candidate genes associated with significant single-nucleotide polymorphisms (SNPs) for plant height and seed coat color.

Trait	SNP Marker	Candidate Gene	Distance to SNP (kb)	Putative Function	Sequence Identity (%)
Plant Height	Chr11_1877114	Sindi.11G025000	12.4	*AP2/ERF* domain-containing protein	95.2
Plant Height	Chr08_1771424	Sindi.08G015600	8.7	Cytochrome P450 *CYP90B1* (Brassinosteroid biosynthesis)	88.7
a*	Chr06_27694080	Sindi.06G123400	15.2	*WRKY* transcription factor 23	94.3
L*	Chr12_16523829	Sindi.12G045200	22.8	Squamosa promoter-binding protein 1	97.8
a*	Chr03_15984975	Sindi.03G078100	18.5	DOF zinc finger protein *DOF3.1*	82.1
a*	Chr03_26242291	Sindi.03G090200	31.7	Serine/threonine-protein kinase STY8	98.5
b*	Chr09_22387055	Sindi.09G078500	26.3	Salicylic acid-binding protein 2	96.7

**Table 4:** Synthesis of major QTL hotspots for sesame seed coat color from previous research.

Chr.	Key QTL Region	PVE Range (%)	Population	Key Candidates	Reference
4	qSCa-4.1, qscca*4 (∼78–81 cm)	8.56–23.10	RIL, F_3_	*DIR* gene family	[26,29]
6	qBSCchr6 (∼2.1 cm)	Major QTL	RIL (BSA)	13 candidate brown seed locus	[32]
6	Meta-QTL hotspot	8.50–25.50	Meta-analysis	*PPO*, *WRKY* TFs	This study (Table 1)
9	qsccY9, qsccZ9 (∼90–104 cm)	32.88–33.25	F_3_	*MYB*, *bHLH* TFs	[26]
9	Meta-QTL hotspot	10.15–32.88	Meta-analysis	*MYB*/*bHLH* complex	Table 1
12	qsccZ12	5.58	F_3_	–	[26]
12, 13	Novel GWAS associations	6.22–6.51	Ethiopian panel	*SBP-like*, Kinase STY8	Table 1

## Data Availability

All supplementary tables, the curated phenotypic dataset, and the filtered SNP dataset (Excel format) for the Ethiopian panel are available in the Figshare repository at [DOI: 10.6084/m9.figshare.31082782]. These data are publicly accessible. The plant materials are maintained by the Ethiopian Biodiversity Institute (EBI), Addis Ababa, and may be requested according to EBI’s material transfer agreements. Public resequencing data used for comparative analysis are available under BioProject PRJNA626474.

## Supplementary Materials

The following supporting information can be downloaded at: https://www.mdpi.com/article/10.3390/plants15030463/s1. Supplementary Table S1. Summary of studies included in the meta-QTL analysis; Supplementary Table S2. Meta-QTL intervals with physical coordinates of candidate genes; Supplementary Table S3. Phenotypic data of Ethiopian sesame accessions for Plant Height and Seed Coat Color Traits (mean of 3 replications); Supplementary Table S4. SNP markers used in Genome-wide Association Study (GWAS); Supplementary Table S5. Complete list of 36 significant SNPs in GWAS for plant height and seed coat color traits; Supplementary Table S6. Annotated candidate genes associated with significant SNPs; Supplementary Table S7. In silico variant analysis of high-priority candidate genes; Supplementary Table S8. List of studies excluded from the meta-QTL analysis with exclusion criteria; Supplementary Table S9. Annotated candidate genes associated with significant SNPs; Supplementary Table S10. Geographical Variation in Phenotypic Traits across Ethiopian regions.

## Abbreviations

AIC	Akaike Information Criterion
AICc	Corrected Akaike Information Criterion
*AP2/ERF*	APETALA2/ETHYLENE RESPONSE FACTOR
BIC	Bayesian Information Criterion
BSA	Bulked Segregant Analysis
BWA-MEM	Burrows–Wheeler Aligner—Maximal Exact Matches (bioinformatics tool)
Chr	Chromosome
cM	Centimorgan
CR-400	Model of the Konica Minolta Chroma Meter
CV	Coefficient of Variation
*CYP90B1*	Cytochrome P450 90B1 (DWF4 gene)
*DIR*	*DIR*igent (gene family)
DOF	DNA-binding One Zinc Finger
EBI	Ethiopian Biodiversity Institute
F_2_	Second Filial Generation
F_3_	Third Filial Generation
F_7_	Seventh Filial Generation
F_8_	Eighth Filial Generation
FAO	Food and Agriculture Organization
FarmCPU	Fixed and Random Model Circulating Probability Unification
FEM	Fixed-Effect Model
*FST*	Fixation Index (population genetic statistic)
GAPIT	Genome Association and Prediction Integrated Tool
GATK	Genome Analysis Toolkit
GBS	Genotyping-by-Sequencing
GFF3	General Feature Format version 3
GWAS	Genome-Wide Association Study
H^2^	Broad-sense Heritability
HTRX	Haplotype Trend Regression with eXclusion
IBS	Identity-by-State
K	Number of genetic clusters (in population structure)
KASP	Kompetitive Allele-Specific PCR
kb	Kilobase
LD	Linkage Disequilibrium
LOD	Logarithm of Odds
LOESS	Locally Estimated Scatterplot Smoothing
L*	Lightness (CIELAB color space parameter)
a*	Green–Red component (CIELAB color space parameter)
b*	Blue–Yellow component (CIELAB color space parameter)
MAF	Minor Allele Frequency
MAS	Marker-Assisted Selection
Mb	Megabase
*MYB*	v-*MYB* avian myeloblastosis viral oncogene homolog (transcription factor family)
*bHLH*	Basic Helix-Loop-Helix (transcription factor family)
NCBI nr	National Center for Biotechnology Information non-redundant (database)
PCA	Principal Component Analysis
PEG	Polyethylene Glycol
PH	Plant Height
PLINK	Whole Genome Association Analysis Toolset
*PPO*	Polyphenol Oxidase
PVE	Phenotypic Variance Explained
Q-matrix	Ancestry proportion matrix (from population structure)
Q–Q plot	Quantile–Quantile plot
QTL	Quantitative Trait Locus/Loci
r	Number of replicates
r^2^	Squared correlation coefficient (measure of LD)
RAD-seq	Restriction-site Associated DNA Sequencing
REM	Random-Effect Model (mentioned as background model in FarmCPU)
RIL	Recombinant Inbred Line
SBP	SQUAMOSA Promoter-Binding Protein
SCC	Seed Coat Color
*SIACS9*	Sesamum indicum 1-aminocyclopropane-1-carboxylic acid synthase 9
*SICEN2*	Sesamum indicum Centroradialis 2
Sindi	Sesamum indicum (gene prefix in genome annotation)
SLAF	Specific-Length Amplified Fragment
SLUBI	Swedish University of Agricultural Sciences Bioinformatics Infrastructure
SLU	Swedish University of Agricultural Sciences
SNP	Single Nucleotide Polymorphism
SSD	Single-Seed Descent
SSR	Simple Sequence Repeat
STY8	Serine/Threonine-protein kinase STY8
TASSEL	Trait Analysis by aSSociation, Evolution and Linkage
TF	Transcription Factor
VCF	Variant Call Format
VCFtools	Variant Call Format tools
WARC	Werer Agricultural Research Center
*WRKY*	Transcription factor family named after the conserved *WRKY* domain
