# Supplementary material for: Integrating Meta-QTL Analysis and Genome-Wide Association Mapping in Ethiopian Sesame (Sesamum indicum L.) Reveals Novel Loci for Plant Height and Seed Coat Color
Source: Plants (Basel). 2026 Feb 2;15(3):463. doi: 10.3390/plants15030463 (PMC12899116; doi:10.3390/plants15030463)
Supplement: Supplementary file 1 [file plants-15-00463-s001.zip › Supplementary Table S1.pdf]

Supplementary Table S1. Summary of studies included in the meta-QTL analysis

Study	Trait(s)	Mapping Method	Population Type	Population Size	Genetic/Physical Map Used	Key Markers	QTL/GWAS Count
[21]	SCC	GWAS	Association panel (366)	366	Physical (v3.0)	3.2M SNPs	12 loci
[26]	PH, SCC	QTL	RIL (F_8, 200 lines)	200	Genetic (SLAF-map)	4,925 SLAFs	8 QTL
[29]	SCC	QTL	F_2 (180 individuals)	180	Genetic (SSR, SNP)	452 markers	5 QTL
[23]	SCC	QTL	F_2 (250 individuals)	250	Genetic (SSR)	187 markers	4 QTL
[32]	PH	QTL	RIL (F_7, 150 lines)	150	Genetic (RAD-seq)	1,256 SNPs	6 QTL
[44]	PH	QTL	RIL (F_8, 188 lines)	188	Genetic (GBS)	2,114 SNPs	5 QTL
[43]	SCC	QTL	RIL (BSA) (150 lines)	150	Genetic (RAD-seq)	1,256 SNPs	6 QTL
[11]	PH, SCC	GWAS	Association panel (280)	280	Physical (v3.0)	2.8M SNPs	10 loci
