# Supplementary material for: Integrating Meta-QTL Analysis and Genome-Wide Association Mapping in Ethiopian Sesame (Sesamum indicum L.) Reveals Novel Loci for Plant Height and Seed Coat Color
Source: Plants (Basel). 2026 Feb 2;15(3):463. doi: 10.3390/plants15030463 (PMC12899116; doi:10.3390/plants15030463)
Supplement: Supplementary file 1 [file plants-15-00463-s001.zip › Supplementary Table S10.pdf]

Supplementary Table S10. Geographical Variation in Phenotypic Traits across Ethiopian regions.

Region	No. Accessions	Mean Plant Height (cm)	Mean L*	Mean a*	Mean b*
Tigray	40	128,5	40,2	4	11,2
Amhara	50	125,1	37,8	3,5	10,8
Oromia	60	130,2	39,5	3,7	11,5
Benishangul Gumuz	30	120,8	35,4	4,1	10,2
Gambella	20	118,6	34,9	3	9,8
