# Supplementary material for: Integrating Meta-QTL Analysis and Genome-Wide Association Mapping in Ethiopian Sesame (Sesamum indicum L.) Reveals Novel Loci for Plant Height and Seed Coat Color
Source: Plants (Basel). 2026 Feb 2;15(3):463. doi: 10.3390/plants15030463 (PMC12899116; doi:10.3390/plants15030463)
Supplement: Supplementary file 1 [file plants-15-00463-s001.zip › Supplementary Table S2.pdf]

Supplementary Table S2. Meta-QTL intervals with physical coordinates of candidate genes.

MQTL	Chromosome	Start (Mb)	End (Mb)	CI (Mb)	Supporting QTLs	Weighted PVE (%)	Candidate Genes
MQTL-PH1	3	25	35	10	5	14.8	*SICEN2, SIACS9*
MQTL-PH2	8	1.77	1.80	0.03	4	18.2	*CYP90B1*
MQTL-PH3	11	1.87	1.90	0.03	4	16.5	*AP2/ERF*
MQTL-SC1	4	45	55	10	6	16.1	*DIR* gene family
MQTL-SC2	6	27.69	27.70	0.01	5	20.5	*PPO, WRKY23*
MQTL-SC3	9	88	92	4	4	21.5	*MYB, bHLH*
