# Supplementary material for: Integrating Meta-QTL Analysis and Genome-Wide Association Mapping in Ethiopian Sesame (Sesamum indicum L.) Reveals Novel Loci for Plant Height and Seed Coat Color
Source: Plants (Basel). 2026 Feb 2;15(3):463. doi: 10.3390/plants15030463 (PMC12899116; doi:10.3390/plants15030463)
Supplement: Supplementary file 1 [file plants-15-00463-s001.zip › Supplementary Table S4.pdf]

Supplementary Table S4. SNP markers used in Genome-wide Association Study (GWAS)

SNP ID	Chromosome	Position (bp)	MAF	Missing rate (%)	HWE p-value
Chr01_384717	1	384717	0.326	6.68	0.384
Chr01_71665	1	71665	0.436	1.49	0.531
Chr01_53001	1	53001	0.046	18.17	0.826
Chr01_312186	1	312186	0.286	1.16	0.454
Chr01_238731	1	238731	0.158	3.86	0.132
Chr01_458058	1	458058	0.482	3.59	0.201
Chr02_42413	2	42413	0.047	17.36	0.676
Chr02_231888	2	231888	0.311	2.12	0.372
Chr02_248506	2	248506	0.214	5.17	0.231
Chr02_141878	2	141878	0.051	6.53	0.963
Chr02_221169	2	221169	0.149	5.67	0.443
Chr02_398439	2	398439	0.468	0.18	0.858
**Chr03_15960455**	**3**	**15960455**	**0.265**	**2.50**	**0.008**
**Chr03_15984975**	**3**	**15984975**	**0.224**	**3.25**	**0.012**
**Chr03_26242291**	**3**	**26242291**	**0.198**	**4.50**	**0.025**
Chr03_198847	3	198847	0.274	18.60	0.822
Chr03_271044	3	271044	0.277	13.98	0.819
Chr03_197710	3	197710	0.047	2.32	0.732
Chr03_189352	3	189352	0.032	0.37	0.313
Chr03_256584	3	256584	0.375	17.05	0.019
Chr04_77364	4	77364	0.451	0.02	0.299
Chr04_203283	4	203283	0.280	7.84	0.746
Chr04_437373	4	437373	0.299	14.17	0.092
Chr05_231165	5	231165	0.152	3.78	0.253
Chr05_400727	5	400727	0.081	15.07	0.868
Chr05_349273	5	349273	0.341	9.47	0.154
**Chr06_27694080**	**6**	**27694080**	**0.185**	**3.25**	**7.84E-07**
Chr06_397132	6	397132	0.434	11.12	0.840

SNP ID	Chromosome	Position (bp)	MAF	Missing rate (%)	HWE p-value
Chr07_436129	7	436129	0.406	3.21	0.595
Chr07_221760	7	221760	0.452	17.58	0.944
Chr07_132744	7	132744	0.248	0.10	0.103
Chr07_361575	7	361575	0.323	16.40	0.854
Chr07_44636	7	44636	0.162	2.57	0.760
Chr07_352579	7	352579	0.053	16.80	0.725
Chr07_336898	7	336898	0.456	15.42	0.204
Chr07_396487	7	396487	0.431	11.20	0.032
**Chr08_1771424**	**8**	**1771424**	**0.215**	**2.80**	**3.89E-06**
Chr08_443816	8	443816	0.132	4.77	0.067
Chr08_176472	8	176472	0.261	19.23	0.160
Chr08_215763	8	215763	0.381	4.57	0.527
Chr08_126953	8	126953	0.275	19.80	0.797
Chr08_255596	8	255596	0.496	3.48	0.563
**Chr09_22387055**	**9**	**22387055**	**0.195**	**4.25**	**0.00148**
Chr09_317172	9	317172	0.245	15.13	0.773
Chr09_460195	9	460195	0.450	15.78	0.715
Chr10_196691	10	196691	0.418	3.15	0.648
Chr10_407710	10	407710	0.498	9.31	0.469
Chr10_440789	10	440789	0.254	1.68	0.868
Chr10_138301	10	138301	0.1847	7.53	0.9096
Chr10_441413	10	441413	0.4450	18.95	0.5437
Chr11_175873	11	175873	0.4799	4.91	0.4465
**Chr11_253662**	**11**	**253662**	**0.2722**	**17.63**	**0.0020**
Chr11_170829	11	170829	0.3755	16.37	0.9646
Chr11_296406	11	296406	0.3570	8.64	0.3214

Note: This table shows 56 SNPs of the 3,683 high-confidence biallelic SNPs used in the GWAS analysis.

SNPs highlighted in **bold** correspond to significant marker-trait associations identified in GWAS.
