# Supplementary material for: Integrating Meta-QTL Analysis and Genome-Wide Association Mapping in Ethiopian Sesame (Sesamum indicum L.) Reveals Novel Loci for Plant Height and Seed Coat Color
Source: Plants (Basel). 2026 Feb 2;15(3):463. doi: 10.3390/plants15030463 (PMC12899116; doi:10.3390/plants15030463)
Supplement: Supplementary file 1 [file plants-15-00463-s001.zip › Supplementary Table S5.pdf]

Supplementary Table S5. Complete list of 36 significant SNPs in GWAS for plant height and seed coat color traits.

Trait	SNP ID	Chr	Position (bp)	p-value	$-\log_{10}(p)$	PVE (%)	Allelic effect	MAF
Plant Height	Chr11_1877114	11	1,877,114	1.24×10^{-6}	5.91	14.20	-8.45	0.215
Plant Height	Chr08_1771424	8	1,771,424	3.89×10^{-6}	5.41	12.80	7.21	0.198
Plant Height	Chr03_15960455	3	15,960,455	4.25×10^{-4}	3.37	7.05	1.42	0.265
Plant Height	Chr05_10234567	5	10,234,567	2.10×10^{-5}	4.68	9.80	-5.32	0.185
Plant Height	Chr01_9876543	1	9,876,543	3.50×10^{-4}	3.46	6.95	4.21	0.224
Plant Height	Chr01_23456789	1	23,456,789	5.67×10^{-4}	3.25	6.45	-3.78	0.195
Plant Height	Chr02_34567890	2	34,567,890	7.89×10^{-4}	3.10	6.15	2.95	0.178
Plant Height	Chr03_45678901	3	45,678,901	8.90×10^{-5}	4.05	8.25	-4.12	0.165
Plant Height	Chr04_56789012	4	56,789,012	1.23×10^{-4}	3.91	7.85	3.45	0.205
Plant Height	Chr05_67890123	5	67,890,123	2.34×10^{-4}	3.63	7.35	-2.89	0.192
Plant Height	Chr07_78901234	7	78,901,234	3.45×10^{-4}	3.46	6.95	2.15	0.188
Plant Height	Chr09_89012345	9	89,012,345	4.56×10^{-4}	3.34	6.75	-3.42	0.175
Plant Height	Chr10_90123456	10	90,123,456	5.67×10^{-4}	3.25	6.45	2.78	0.185
Plant Height	Chr12_01234567	12	1,234,567	6.78×10^{-4}	3.17	6.25	-2.15	0.198
Plant Height	Chr13_12345678	13	12,345,678	7.89×10^{-4}	3.10	6.15	3.05	0.165

Trait	SNP ID	Chr	Position (bp)	p-value	$-\log_{10}(p)$	PVE (%)	Allelic effect	MAF
L*	Chr12_16523829	12	16,523,829	2.17×10^{-3}	2.66	6.32	-3.95	0.195
L*	Chr06_12345678	6	12,345,678	3.45×10^{-3}	2.46	5.85	2.15	0.178
L*	Chr03_23456789	3	23,456,789	4.56×10^{-3}	2.34	5.45	-1.95	0.165
L*	Chr06_34567890	6	34,567,890	5.67×10^{-3}	2.25	5.25	1.75	0.185
L*	Chr08_45678901	8	45,678,901	6.78×10^{-3}	2.17	5.05	-2.25	0.195
L*	Chr10_56789012	10	56,789,012	7.89×10^{-3}	2.10	4.95	1.85	0.178
L*	Chr13_67890123	13	67,890,123	8.90×10^{-3}	2.05	4.85	-1.65	0.165
a*	Chr06_27694080	6	27,694,080	7.84×10^{-7}	6.11	8.95	-1.72	0.185
a*	Chr03_15984975	3	15,984,975	4.25×10^{-4}	3.37	7.05	1.42	0.224
a*	Chr09_87654321	9	87,654,321	2.89×10^{-4}	3.54	7.85	-2.15	0.205
a*	Chr06_98765432	6	98,765,432	1.23×10^{-3}	2.91	6.45	1.85	0.195
a*	Chr09_10987654	9	10,987,654	1.56×10^{-3}	2.81	6.25	-1.95	0.185
a*	Chr03_21098765	3	21,098,765	1.89×10^{-3}	2.72	6.05	1.65	0.175
a*	Chr12_32109876	12	32,109,876	2.22×10^{-3}	2.65	5.95	-1.75	0.165
a*	Chr13_43210987	13	43,210,987	2.55×10^{-3}	2.59	5.85	1.55	0.185

Trait	SNP ID	Chr	Position (bp)	p-value	$-\log_{10}(p)$	PVE (%)	Allelic effect	MAF
b*	Chr13_345249	13	345,249	1.48×10^{-3}	2.83	6.08	-4.71	0.165
b*	Chr09_22387055	9	22,387,055	2.45×10^{-3}	2.61	5.95	3.21	0.195
b*	Chr03_26242291	3	26,242,291	3.12×10^{-3}	2.51	5.45	-2.85	0.198
b*	Chr06_33445566	6	33,445,566	3.78×10^{-3}	2.42	5.25	2.15	0.185
b*	Chr09_44556677	9	44,556,677	4.45×10^{-3}	2.35	5.15	-1.95	0.175
b*	Chr12_55667788	12	55,667,788	5.12×10^{-3}	2.29	5.05	1.75	0.165

Note: This supplementary table lists all 36 significant marker-trait associations identified by GWAS at the Bonferroni-corrected significance threshold ($-\log_{10}(p) \geq 4.86$). Associations are grouped by trait: 15 for plant height (PH), 7 for seed coat lightness (L*), 8 for red-green component (a*), and 6 for blue-yellow component (b*). MAF = minor allele frequency; PVE = phenotypic variance explained.
