# Supplementary material for: Integrating Meta-QTL Analysis and Genome-Wide Association Mapping in Ethiopian Sesame (Sesamum indicum L.) Reveals Novel Loci for Plant Height and Seed Coat Color
Source: Plants (Basel). 2026 Feb 2;15(3):463. doi: 10.3390/plants15030463 (PMC12899116; doi:10.3390/plants15030463)
Supplement: Supplementary file 1 [file plants-15-00463-s001.zip › Supplementary Table S6.pdf]

Supplementary Table S6. Annotated candidate genes associated with significant SNPs.

Significant SNP	Chromosome	Gene ID	Start (bp)	End (bp)	Annotation	Sequence identity (%)
Chr11_1877114	11	Sindi.11G025000	1,870,000	1,875,000	AP2/ERF domain-containing protein	95.2
Chr08_1771424	8	Sindi.08G015600	1,770,000	1,775,000	Cytochrome P450 *CYP90B1*	88.7
Chr06_27694080	6	Sindi.06G123400	27,690,000	27,695,000	*WRKY* transcription factor 23	94.3
Chr12_16523829	12	Sindi.12G045200	16,520,000	16,525,000	Squamosa promoter-binding protein 1	97.8
Chr03_15984975	3	Sindi.03G078100	15,984,000	15,989,000	DOF zinc finger protein *DOF3.1*	82.1
Chr03_15960455	3	Sindi.03G090200	15,960,000	15,965,000	Serine/threonine-protein kinase STY8	98.5
Chr09_22387055	9	Sindi.09G078500	22,386,000	22,391,000	Salicylic acid-binding protein 2	96.7
Chr13_345249	13	Sindi.13G005500	340,000	350,000	*SBP-like* transcription factor	92.4
