# Supplementary material for: Integrating Meta-QTL Analysis and Genome-Wide Association Mapping in Ethiopian Sesame (Sesamum indicum L.) Reveals Novel Loci for Plant Height and Seed Coat Color
Source: Plants (Basel). 2026 Feb 2;15(3):463. doi: 10.3390/plants15030463 (PMC12899116; doi:10.3390/plants15030463)
Supplement: Supplementary file 1 [file plants-15-00463-s001.zip › Supplementary Table S7.pdf]

Supplementary Table S7. In silico variant analysis of high-priority candidate genes.

Gene ID	Chromosome	SNP/InDEL ID	Variant type	Location	Alleles (Ref/Alt)	MAF in Panel	Phenotypic association	Predicted effect	Notes
Sindi.08G015600 (*CYP90B1*)	8	Chr08_1771424	SNP	CDS (exon 3)	C/T	0.215	Plant height (PH)	Non-synonymous (Pro → Ser)	Co-segregates with dwarf phenotype
Sindi.11G025000 (AP2/ERF)	11	Chr11_1877114	SNP	Promoter (−287 bp)	A/G	0.198	Plant height (PH)	TF-binding site alteration	Higher expression in tall accessions
Sindi.06G123400 (*WRKY23*)	6	Chr06_27694080	SNP	CDS (exon 2)	G/A	0.185	Seed coat color (a*)	Non-synonymous (Arg → Lys)	Associated with darker seeds
Sindi.03G078100 (*DOF3.1*)	3	Chr03_15984975	SNP	Promoter (−112 bp)	T/C	0.224	Seed coat color (a*)	Light-responsive element	Co-segregates with high a* values
Sindi.12G045200 (*SBP-like*)	12	Chr12_16523829	SNP	CDS (exon 1)	A/T	0.195	Seed coat color (L*)	Synonymous	Linked to light seed coat
Sindi.03G090200 (STY8)	3	Chr03_26242291	INDEL	CDS (exon 4)	+3 bp	0.198	Seed coat color (b*)	In-frame insertion	May affect kinase activity
Sindi.09G078500 (SABP2)	9	Chr09_22387055	SNP	3′ UTR	C/T	0.195	Seed coat color (b*)	Possible mRNA stability effect	Associated with high b* values

Notes: CDS = coding sequence; UTR = untranslated region; MAF = minor allele frequency. Variants were called from resequencing data (BioProject PRJNA626474) using GATK best practices. Promoter regions are defined as 1 kb upstream of the transcription start site. Phenotypic extremes were defined as the top/bottom 10% of trait distribution.
