# Supplementary material for: Integrating Meta-QTL Analysis and Genome-Wide Association Mapping in Ethiopian Sesame (Sesamum indicum L.) Reveals Novel Loci for Plant Height and Seed Coat Color
Source: Plants (Basel). 2026 Feb 2;15(3):463. doi: 10.3390/plants15030463 (PMC12899116; doi:10.3390/plants15030463)
Supplement: Supplementary file 1 [file plants-15-00463-s001.zip › Supplementary Table S8.pdf]

Supplementary Table S8. List of studies excluded from the meta-QTL analysis with exclusion criteria.

Study (Author, Year)	Trait(s)	Reason for exclusion	Specific issue
Zhang et al., 2015	PH, SCC	Incomplete mapping data	Missing LOD scores or PVE values
Li et al., 2017	SCC	Population not clearly defined	Used mixed F_2/F_3 without a clear pedigree
Chen et al., 2018	PH	Marker positions were not anchorable	Used legacy SSR markers without genome coordinates
Kumar et al., 2019	SCC	Overlapping population with the included study	Same RIL population as [26]
Wang et al., 2020	PH, SCC	GWAS without QTL intervals	Reported only SNP p-values, no QTL intervals
Singh et al., 2021	SCC	Trait measurement inconsistent	Used subjective color scoring, not CIELAB
Patel et al., 2022	PH	Low mapping resolution	≤5 markers per chromosome
Yao et al., 2023	SCC	Unpublished genetic map	Used a custom linkage map not publicly available
Ahmed et al., 2024	PH	Data not in English	Full text not accessible in English
Garcia et al., 2024	SCC	Conference abstract only	Insufficient methodological detail

Exclusion criteria applied: Missing essential QTL parameters (LOD, PVE, confidence interval); marker systems not mappable to reference genome v3.0; population overlaps with already included studies; trait measurement not comparable (non-CIELAB color scoring); and incomplete or non-peer-reviewed data.
