# Supplementary material for: Integrating Meta-QTL Analysis and Genome-Wide Association Mapping in Ethiopian Sesame (Sesamum indicum L.) Reveals Novel Loci for Plant Height and Seed Coat Color
Source: Plants (Basel). 2026 Feb 2;15(3):463. doi: 10.3390/plants15030463 (PMC12899116; doi:10.3390/plants15030463)
Supplement: Supplementary file 1 [file plants-15-00463-s001.zip › Supplementary Table S9.pdf]

Supplementary Table S9. Annotated candidate genes associated with significant SNPs

Category	Term	Description	Gene count	*P*-value	FDR
GO: Biological Process	Brassinosteroid biosynthesis	Enrichment of BR-related genes, including CYP90B1	3	0,004	0,01
GO: Molecular Function	Transcription factor activity	AP2/ERF, WRKY23, DOF3.1 involved in gene regulation	4	0,012	0,03
GO: Cellular Component	Nucleus	Candidate genes localized to nucleus	6	0,020	0,05
KEGG Pathway	Plant hormone signal transduction	BR, auxin, ethylene signaling pathways	5	0,008	0,02
KEGG Pathway	Phenylpropanoid biosynthesis	Pigmentation and flavonoid pathways for seed coat color	3	0,015	0,04
